# Supplementary material for: The enzymatic activity of inositol hexakisphosphate kinase controls circulating phosphate in mammals
Source: Nat Commun. 2021 Aug 11;12:4847. doi: 10.1038/s41467-021-24934-8 (PMC8358040; doi:10.1038/s41467-021-24934-8)
Supplement: Supplementary file 1 — Supplementary Information [file 41467_2021_24934_MOESM1_ESM.pdf]

Supplementary information

The enzymatic activity of inositol hexakisphosphate kinase controls circulating phosphate in mammals

Yusuke Moritoh[1*], Shin-ichi Abe[1], Hiroki Akiyama[1], Akihiro Kobayashi[1], Ryokichi Koyama[1], Ryoma Hara[1], Shizuo Kasai[1], Masanori Watanabe[1]

[1]Research Division, SCOHIA PHARMA Inc., Kanagawa, Japan

[*]Correspondence to: yusuke.moritoh@scohia.com

^1H NMR

Acquisition Time (sec)	3.9846	*Date*	18 Apr 2014 19:03:15
Date Stamp	18 Apr 2014 19:03:15		
File Name	\\fs26\medchem\data\C3031093\nmr\D18709-23-001\10\PDATA\1\1r		
Frequency (MHz)	400.13	*Nucleus*	1H
Number of Transients	16	*Origin*	spect
Original Points Count	32768	*Owner*	root
Points Count	65536	*Pulse Sequence*	zg30
Receiver Gain	164.67	*SW(cyclical) (Hz)*	8223.68
Solvent	DMSO-d6	*Spectrum Offset (Hz)*	2468.2964
Spectrum Type	STANDARD	*Sweep Width (Hz)*	8223.56
Temperature (degree C)	28.378		

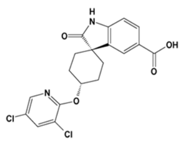

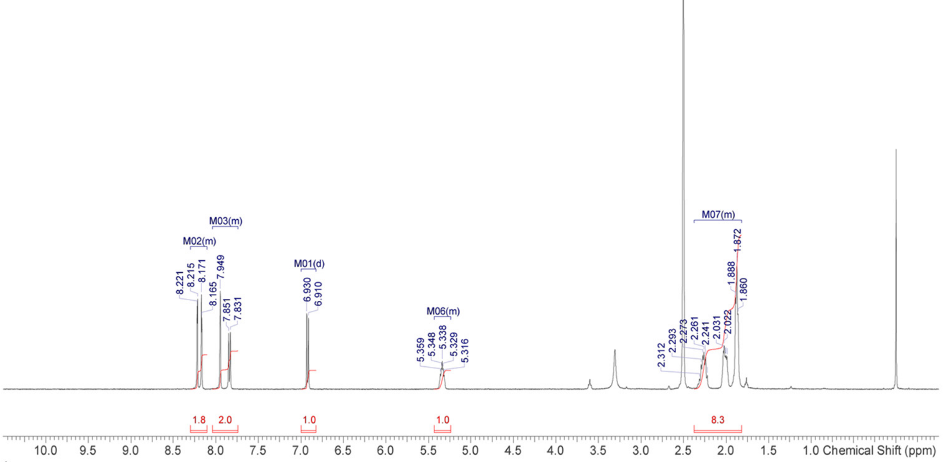

^1H NMR (400 MHz, DMSO-d_6) δ 1.82-2.38 (8H, m), 5.24-5.43 (1H, m), 6.92 (1H, d, J = 8.2 Hz), 7.74-8.04 (2H, m), 8.10-8.30 (2H, m), 10.70 (1H, s), 12.62 (1H, brs).

^{13}C NMR

2021/03/26 13:36:51

Number of Nuclei 17 C's / 19 C's (spectrum / structure)	*Multiplets Integrals Sum* 0.00	*Low Signal to Noise Ratio* True	
Formula $C_{19}H_{16}Cl_2N_2O_4$	*FW* 407.2473	*MF (Multi Spec)* 0.92	

Acquisition Time (sec)	1.8088	*Comment*	HK7868-001r			*D*	0.03	*D1*	2
DE	6.5	*DS*	4	*Date*	04 Mar 2021 08:22:36 (GMT+09:00)			*Date Stamp*	04 Mar 2021 08:22:36 (GMT+09:00)
ExpNo	10	*File Name*	E:¥data¥HK7868-001r¥10¥pdata¥1¥1r			*Frequency (MHz)*	75.4678	*GB*	0
INSTRUM	<spect>	*LB*	1	*NS*	8192	*Nucleus*	13C	*Number of Transients*	8192
Origin	spect	*Original Points Count*	32768	*Owner*	nmrsu	*PC*	1.4		
PROBHD	<Z104275_0205 (PA BBO 300S1 BBF-H-D-05 Z)>			*PULPROG*	<zgpg30>	*Points Count*	32768	*Pulse Sequence*	zgpg30
Receiver Gain	196.79	*SF*	75.4678302311318			*SFO1*	75.475295301		
SI	32768	*SSB*	0	*SW(cyclical) (Hz)*	18115.94	*SWH*	18115.9420289855		
Solvent	DMSO-d6	*Spectrum Offset (Hz)*	7503.0664	*Spectrum Type*	standard	*Sweep Width (Hz)*	18115.39	*TD*	65536
TD0	1	*TE*	296.0964	*Temperature (degree C)*	23.096	*UNC1*	<13C>	*WDW*	1

User Notes HK7868-001r

^{13}C NMR (75 MHz, DMSO-d_6) δ ppm 25.35 (s, 1 C) 29.87 (s, 1 C) 45.11 (s, 1 C) 73.44 (s, 1 C) 108.84 (s, 1 C) 117.98 (s, 1 C) 122.69 (s, 1 C) 123.76 (s, 1 C) 124.01 (s, 1 C) 130.11 (s, 1 C) 134.76 (s, 1 C) 138.35 (s, 1 C) 143.18 (s, 1 C) 145.32 (s, 1 C) 156.77 (s, 1 C) 167.20 (s, 1 C) 181.26 (s, 1 C)

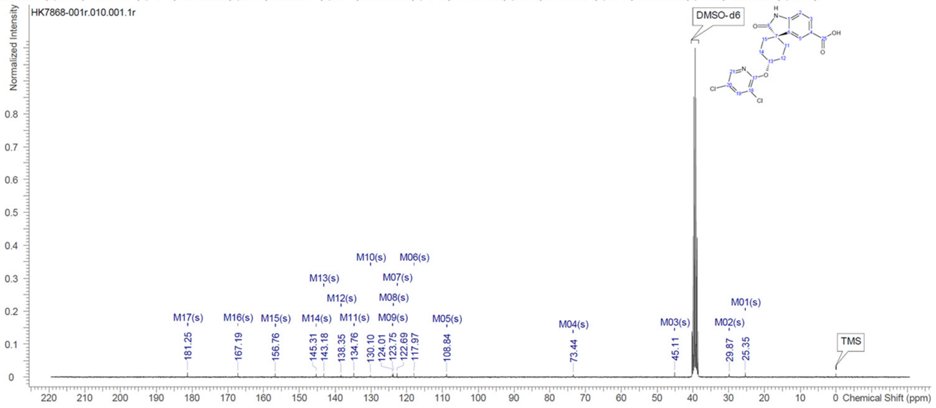

Elemental analysis, Anal. Calcd. for $C_{19}H_{16}N_2O_4Cl_2$: C, 56.04; H, 3.96; N, 6.88. Found: C, 55.67; H, 4.12; N, 7.10.

HRMS, (ESI) *m/z* $(M+H)^+$: Calcd. for $C_{19}H_{17}Cl_2N_2O_4$: 407.0560, Found: 407.0560.

Supplementary Fig. 1. Spectrum data (^1H NMR and ^{13}C NMR), elemental analysis, and HRMS of SC-919.

Supplementary Table 1. Results of the kinome assay for SC-919.

DiscoveRx Gene Symbol	Entrez Gene Symbol	Percent Control	SC-919 Concentration (nM)
ABL1 (E255K)-phosphorylated	ABL1	84	1,000
ABL1 (T315I)-phosphorylated	ABL1	100	1,000
ABL1-nonphosphorylated	ABL1	44	1,000
ABL1-phosphorylated	ABL1	73	1,000
ACVR1B	ACVR1B	100	1,000
ADCK3	CABC1	78	1,000
AKT1	AKT1	90	1,000
AKT2	AKT2	100	1,000
ALK	ALK	100	1,000
AURKA	AURKA	100	1,000
AURKB	AURKB	100	1,000
AXL	AXL	99	1,000
BMPR2	BMPR2	100	1,000
BRAF	BRAF	98	1,000
BRAF (V600E)	BRAF	98	1,000
BTK	BTK	100	1,000
CDK11	CDK19	100	1,000
CDK2	CDK2	100	1,000
CDK3	CDK3	97	1,000
CDK7	CDK7	100	1,000
CDK9	CDK9	100	1,000
CHEK1	CHEK1	100	1,000
CSF1R	CSF1R	99	1,000
CSNK1D	CSNK1D	95	1,000
CSNK1G2	CSNK1G2	100	1,000
DCAMKL1	DCLK1	95	1,000
DYRK1B	DYRK1B	53	1,000
EGFR	EGFR	100	1,000
EGFR (L858R)	EGFR	100	1,000
EPHA2	EPHA2	82	1,000
ERBB2	ERBB2	58	1,000
ERBB4	ERBB4	100	1,000
ERK1	MAPK3	100	1,000
FAK	PTK2	100	1,000
FGFR2	FGFR2	85	1,000
FGFR3	FGFR3	87	1,000
FLT3	FLT3	99	1,000
GSK3B	GSK3B	98	1,000
IGF1R	IGF1R	100	1,000

IKK-alpha	CHUK	100	1,000
IKK-beta	IKBKB	100	1,000
INSR	INSR	82	1,000
JAK2 (JH1 domain-catalytic)	JAK2	100	1,000
JAK3 (JH1 domain-catalytic)	JAK3	78	1,000
JNK1	MAPK8	87	1,000
JNK2	MAPK9	100	1,000
JNK3	MAPK10	100	1,000
KIT	KIT	64	1,000
KIT (D816V)	KIT	94	1,000
KIT (V559D, T670I)	KIT	100	1,000
LKB1	STK11	100	1,000
MAP3K4	MAP3K4	90	1,000
MAPKAPK2	MAPKAPK2	100	1,000
MARK3	MARK3	100	1,000
MEK1	MAP2K1	97	1,000
MEK2	MAP2K2	87	1,000
MET	MET	91	1,000
MKNK1	MKNK1	93	1,000
MKNK2	MKNK2	100	1,000
MLK1	MAP3K9	100	1,000
p38-alpha	MAPK14	100	1,000
p38-beta	MAPK11	95	1,000
PAK1	PAK1	100	1,000
PAK2	PAK2	100	1,000
PAK4	PAK4	100	1,000
PCTK1	CDK16	100	1,000
PDGFRA	PDGFRA	96	1,000
PDGFRB	PDGFRB	62	1,000
PDPK1	PDPK1	95	1,000
PIK3C2B	PIK3C2B	96	1,000
PIK3CA	PIK3CA	96	1,000
PIK3CG	PIK3CG	78	1,000
PIM1	PIM1	100	1,000
PIM2	PIM2	100	1,000
PIM3	PIM3	100	1,000
PKAC-alpha	PRKACA	100	1,000
PLK1	PLK1	95	1,000
PLK3	PLK3	99	1,000
PLK4	PLK4	100	1,000
PRKCE	PRKCE	100	1,000
RAF1	RAF1	90	1,000
RET	RET	99	1,000

RIOK2	RIOK2	100	1,000
ROCK2	ROCK2	100	1,000
RSK2 (Kin.Dom.1-N-terminal)	RPS6KA3	62	1,000
SNARK	NUAK2	100	1,000
SRC	SRC	100	1,000
SRPK3	SRPK3	94	1,000
TGFBR1	TGFBR1	94	1,000
TIE2	TEK	97	1,000
TRKA	NTRK1	100	1,000
TSSK1B	TSSK1B	98	1,000
TYK2 (JH1 domain-catalytic)	TYK2	74	1,000
ULK2	ULK2	100	1,000
VEGFR2	KDR	84	1,000
YANK3	STK32C	96	1,000
ZAP70	ZAP70	100	1,000

Percent Control: The compound was screened at the indicated concentration, and results for primary screen binding interactions are reported as % Control. The % Control calculation was performed as: (test compound signal – positive control signal)/(negative control signal – positive control signal) × 100. Test compound = SC-919, negative control = DMSO (100 % Control), positive control = control compound (0 % Control).

a InsP$_6$-d_6: ^{31}P NMR spectrum (162 MHz, D_2O, 25 ℃)

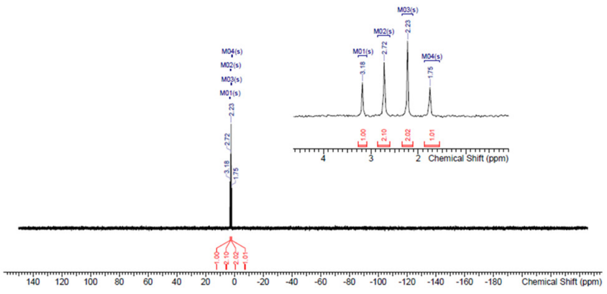

ID2876-001
^{31}P NMR (162 MHz, D_2O, 298 K) δ 1.75 (1P, s), 2.23 (2P, s), 2.72 (2P, s), 3.18 (1P, s).

InsP$_7$: ^{1}H NMR spectrum (500 MHz, D_2O, 25 ℃)

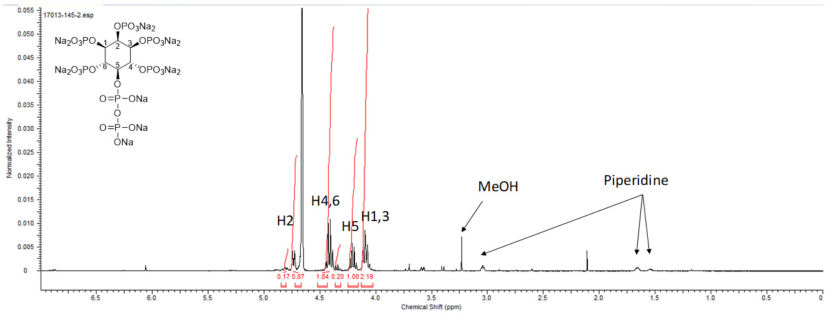

InsP$_7$: ESI-MS m/z 368.92 $[M-2H]^{2-}$(SCIEX TripleTOF™6600, MeOH/H_2O, Negative ion mode)

b

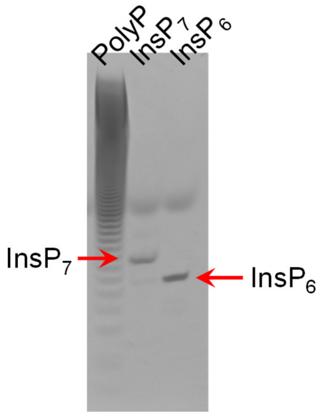

Supplementary Fig. 2. Analytical data of InsP$_6$-d_6, and InsP$_7$ (5-InsP$_7$), and toluidine blue staining of InsP$_7$ and InsP$_6$ following Poly-Acrylamide Gel Electrophoresis (33.3%).
^{31}P NMR spectra (85% H_3PO_4 in D_2O standard) of InsP$_6$-d_6 and ^{1}H NMR spectra and ESI-MS data of InsP$_7$ (a). Electrophoresis of InsP$_7$, InsP$_6$ and PolyP (106529, Supelco; PolyP ladder was used as the electrophoresis standard to maintain the quality of separation) were performed Poly-Acrylamide Gel Electrophoresis (33.3%) and each compound was stained with toluidine blue (b).

Supplementary Table 2. Linear regression parameters using the weighting factor $1/x^2$ for the calibration curves of $InsP_6$ and $InsP_7$ in plasma.

Analyte	Calibration Curve	r	Linear range (ng/mL) [1]	Accuracy (%)
$InsP_6$	y = 1.24x + 0.0564	0.9994	40 – 40,000	95.8 – 104.9
$InsP_7$	y = 0.00159x-0.000117	0.9995	0.2 - 200	96.3 – 104.1

[1]calculated as sodium salt.

Supplementary Table 3. Accuracy and precision of $InsP_6$ in quality control samples (n=3).

Matrix	QC	Concentration（ng/mL）[1]	Accuracy（%）[2]	Precision（%）[3]
Liver	LQC	6,000	98.4	1.6
	MQC	20,000	93.5	2.2
	HQC	32,000	91.1	2.5
Muscle	LQC	1,000	101.2	0.6
	MQC	10,000	97.7	3.5
	HQC	20,000	98.2	0.9
Kidney	LQC	6,000	97.6	3.3
	MQC	20,000	93.3	2.2
	HQC	32,000	90.9	1.7
293 cells	LQC	400	99.7	0.3
	MQC	4,000	101.2	1.1
	HQC	10,000	97.3	1.7
HAP1 cells	LQC	1,000	96.9	2.6
	MQC	10,000	94.9	3.5
	HQC	20,000	96.4	1.9

[1]calculated as sodium salt.
[2]calculated as (mean determined amount / nominal amount × 100).
[3]calculated as % CV (SD / mean) × 100.

Supplementary Table 4. Accuracy and precision of $InsP_7$ in quality control samples (n = 3).

Matrix	QC	Concentration （ng/mL）[1]	Accuracy（%）[2]	Precision（%）[3]
Liver	LQC	0.6	100.1	2.4
	MQC	10	97.4	1.8
	HQC	160	83.2	0.8
Muscle	LQC	0.2	99.1	1.9
	MQC	4	95.5	0.7
	HQC	20	99.1	2.7
Kidney	LQC	6	96.0	2.5
	MQC	40	90.8	2.2
	HQC	160	90.9	2.6
	DIQC	400	104.3	1.8
293 cells	LQC	0.6	101.4	3.8
	MQC	4	104.9	1.8
	HQC	40	101.5	1.4
HAP1 cells	LQC	0.4	118.0	9.3
	MQC	2	111.0	1.8
	HQC	20	106.0	3.8

[1]calculated as sodium salt.
[2]calculated as (mean determined amount / nominal amount × 100).
[3]calculated as % CV (SD / mean) × 100.

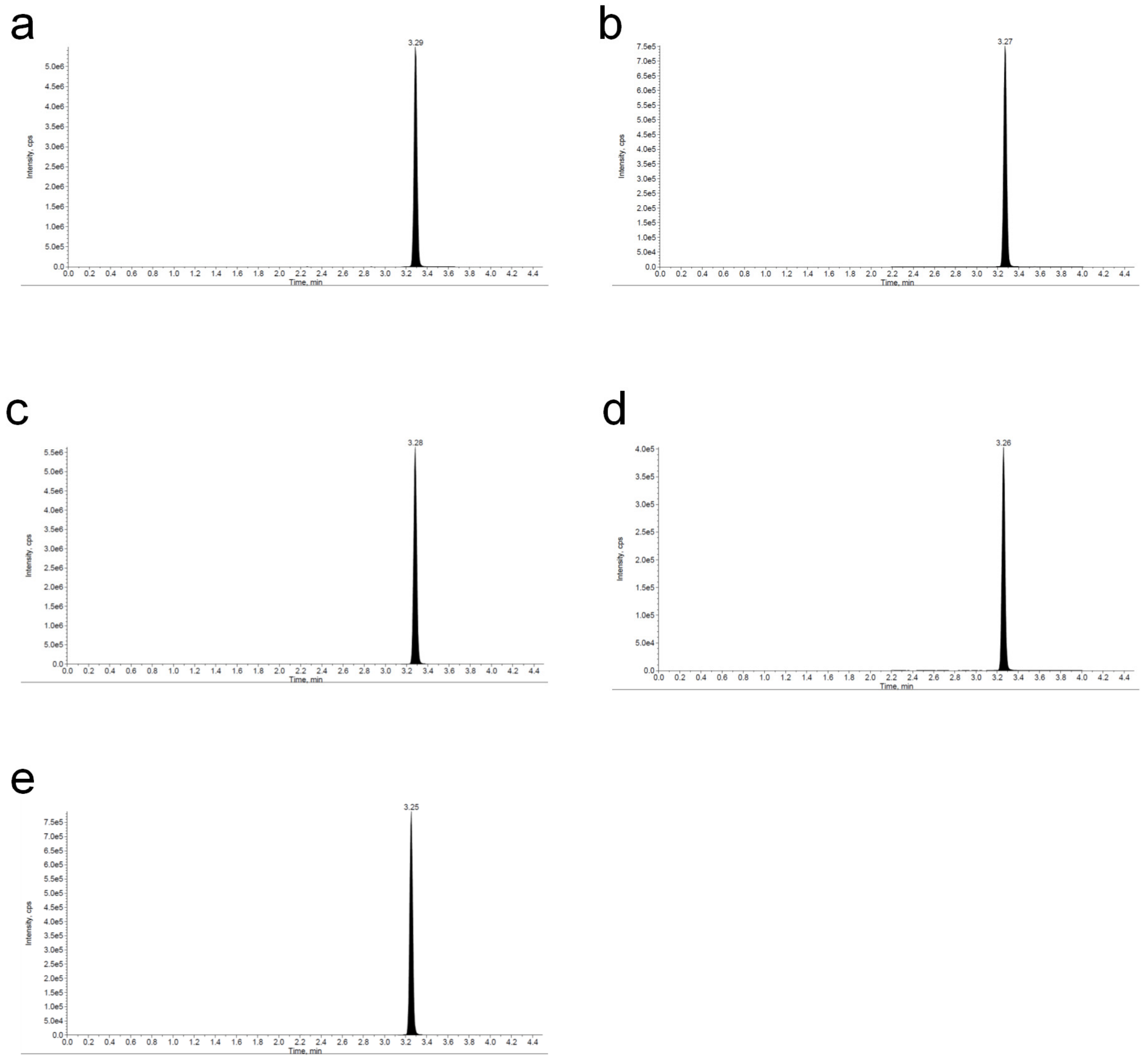

Supplementary Fig. 3. Representative chromatograms at LQC of $InsP_6$ in a) liver, b) muscle, c) kidney, d) 293 cells, and e) HAP1 cells.

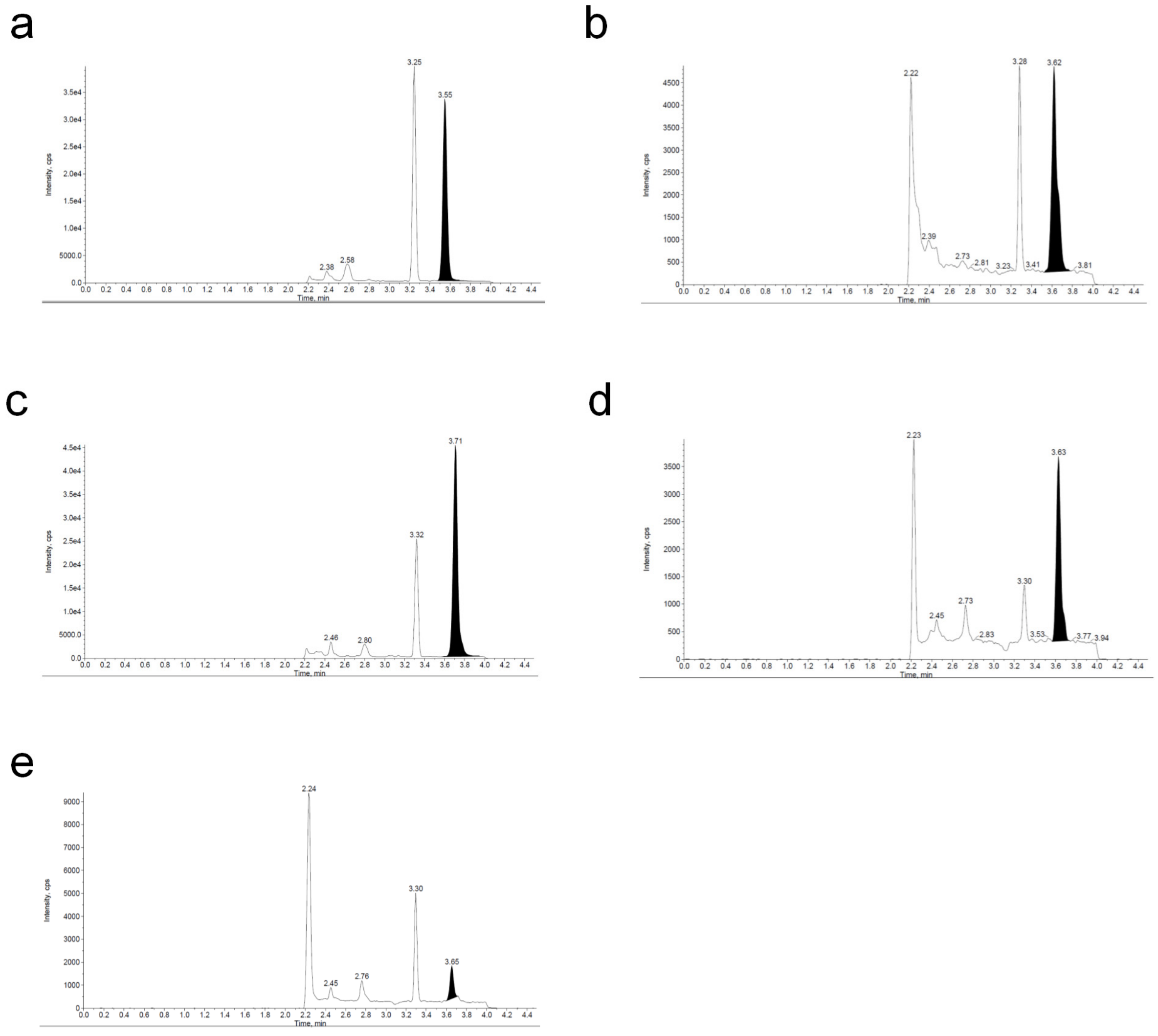

Supplementary Fig. 4. Representative chromatograms at LQC of InsP$_7$ in a) liver, b) muscle, c) kidney, d)293 cells, and e) HAP1 cells.

Supplementary Table 5. Tissue concentration of SC-919.

Dose level	Tissue concentration of SC-919 (ng/g tissue)		
	Liver	Muscle	Kidney
SC-919 1 mg/kg	362 ± 53	128 ± 36	668 ± 135
SC-919 10 mg/kg	1979 ± 87	1051± 226	3152 ± 347

SC-919 was administered to 8-week-old SD rats, and each tissue sample was collected 2 h post dosing with SC-919. Values indicate mean ± S.D. (n = 6 biological replicates)

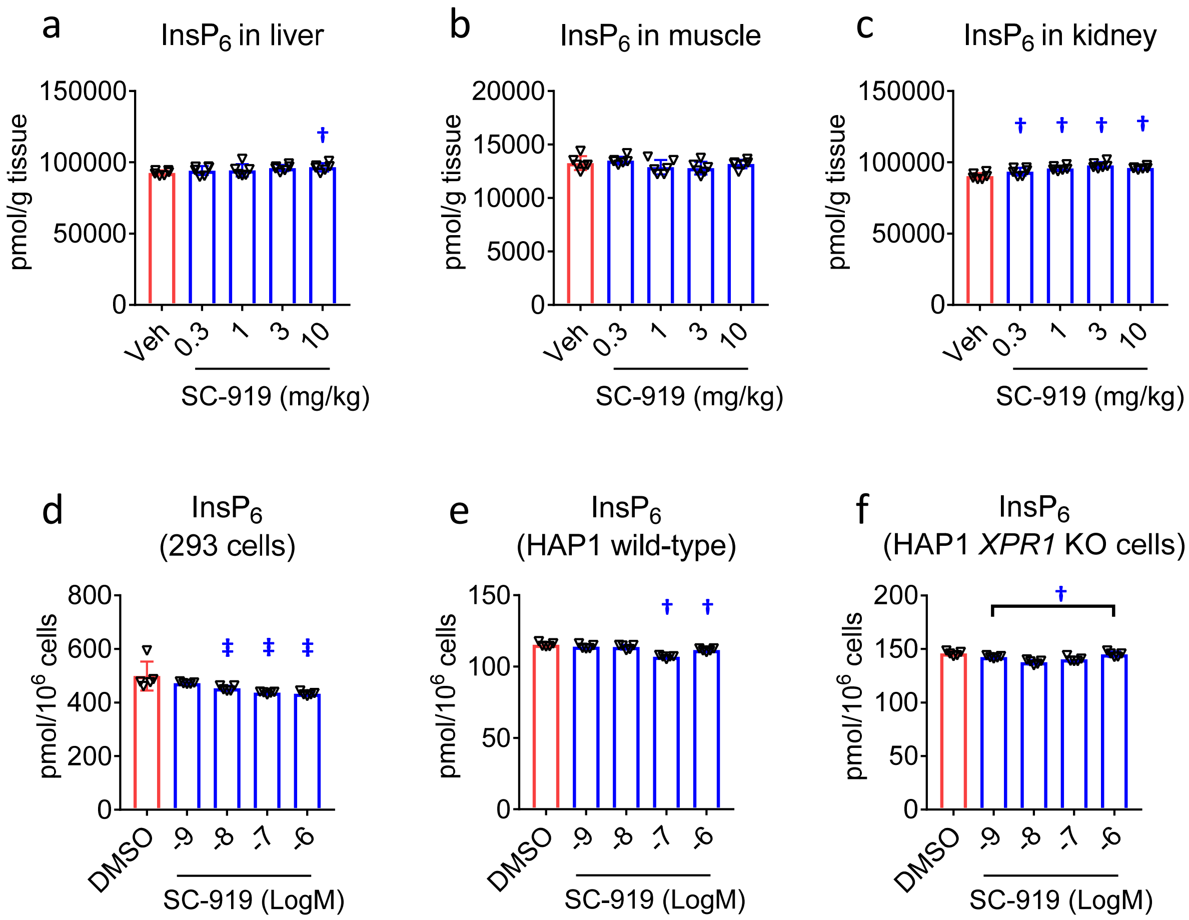

Supplementary Fig. 5. $InsP_6$ levels in tissues and cells. (a, b, c) $InsP_6$ levels in the liver, skeletal muscles, and kidney 2 h after the administration of SC-919 to SD rats (n = 6 biological replicates). (d, e, f) $InsP_6$ levels in 293, HAP1 wild-type, and HAP1 *XPR1* KO cells treated with SC-919 for 4 h (n = 5 biological replicates). Values indicate mean ± S.D. †*P* < 0.05 and ‡*P* < 0.05 vs. vehicle or DMSO as determined using Williams' test and Shirley–Williams test, respectively. Veh, vehicle.

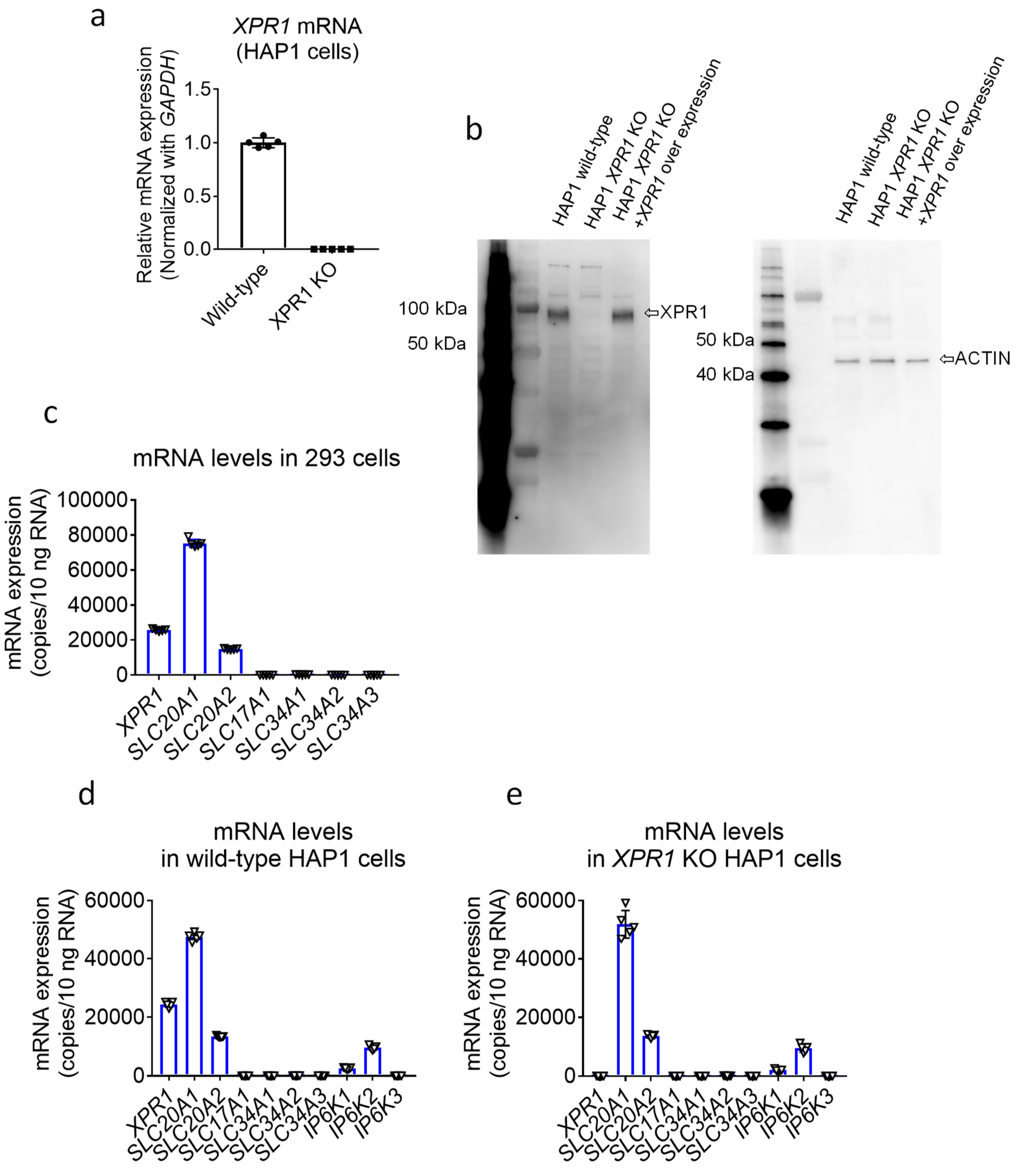

Supplementary Fig. 6. Gene and protein expression profiles in cells. (a) Levels of *XPR1* mRNA in HAP1 wild-type and *XPR1* KO cells. (b) Levels of XPR1 protein in HAP1 wild-type and *XPR1* KO cells. (c, d, e) transcript levels of the indicated genes in 293, HAP1 wild-type, and *XPR1* KO cells. Values indicate mean ± S.D. (n = 5 biological replicates).

Supplementary Table 6. Protein levels in cells treated with SC-919.

Group	293	HAP1 wild-type	HAP1 *XPR1* KO
DMSO	151 ± 24	137 ± 54	130 ± 41
SC-919 0.001 μM	154 ± 34	131 ± 34	131 ± 43
SC-919 0.01 μM	140 ± 37	135 ± 43	126 ± 38
SC-919 0.1 μM	136 ± 48	128 ± 41	132 ± 33
SC-919 1 μM	141 ± 52	115 ± 20	136 ± 26

Values indicate mean ± S.D. (μg/mL/well, n = 6 biological replicates).

Supplementary Table 7. MRM parameters of $InsP_6$, $InsP_7$ and $InsP_6$-d_6.

Analyte	Precursor ion (*m/z*)	Product ion (*m/z*)	DP (V)	EP (V)	CE (V)	CXP (V)
$InsP_6$	658.8	560.8	-80	-10	-37.6	-25
$InsP_7$	738.8	640.8	-80	-10	-37.3	-25
$InsP_6$-d_6	664.7	566.8	-80	-10	-37.6	-25

DP, declustering potential; EP, entrance potential; CE, collision energy; CXP, collision cell exit potential.

Supplementary Table 8. Primers, probes, and standard oligonucleotide sequences used in the probe-based qPCR (Results are shown in Supplementary Fig. 6).

Sequence Name	Sequence (5′→3′)
Hs_SLC20A1-P	TTAGGCAACTGCACTGCACCATTCACGG
Hs_SLC20A1-F	CAGCGTGGACTTGAAAGAGGAA
Hs_SLC20A1-R	GACGGCTTGACTGAACTGGAC
Hs_SLC20A2-P	CTCACTCCCATCACGCCGTCCAGC
Hs_SLC20A2-F	GGAGAAGAGTGATCCAGACCATG
Hs_SLC20A2-R	CACCACTGTGAAGGCTGAGG
Hs_SLC17A1-P	ACAGAACGAACCAGAGAAGACATACGGCAC
Hs_SLC17A1-F	GGTCTTCTATATTTTTGGTGCTTGTG
Hs_SLC17A1-R	GGGTGGTCTTTGGGGTCATC
Hs_SLC34A1-P	CCTCAGGGACTCATCACCAGTGGCAATG
Hs_SLC34A1-F	GCTCATCATCCAGCTGGACG
Hs_SLC34A1-R	CTGTAAGGAGTCTGGGTGGC
Hs_SLC34A2-P	ACAAGGAGAACATCGCCAAATGCCAGC
Hs_SLC34A2-F	ACTGGACCATGAAGAATGTGACC
Hs_SLC34A2-R	CAGCAAGATCCGGGAGGTG
Hs_SLC34A3-P	CACAGGCAACGCCACTAACAGCAGTCTC
Hs_SLC34A3-F	CGTGCAGTTGGACTCCGA

Hs_SLC34A3-R	TGTTCTCCTGGGTCGGCTG
Hs_XPR1-P	AGAAGGCTCAGGCACCACAATATCCCGA
Hs_XPR1-F	GTCTCATCAACATCTCTTTGAGATTG
Hs_XPR1-R	TGTGGGGATGACACTAATTGGAG
Hs_SLC20A1-std	CTTCCCAGCGTGGACTTGAAAGAGGAAACCAGCATAGA TAGCACCGTGAATGGTGCAGTGCAGTTGCCTAATGGGA ACCTTGTCCAGTTCAGTCAAGCCGTCAGCAA
Hs_SLC20A2-std	CTGGGGGAGAAGAGTGATCCAGACCATGGGGAAGGAC CTCACTCCCATCACGCCGTCCAGCGGCTTCACGATCGA GCTGGCCTCAGCCTTCACAGTGGTGATCGC
Hs_SLC17A1-std	CCCATGGTCTTCTATATTTTGGTGCTTGTGGCTGTGCC GTATGTCTTCTCTGGTTCGTTCTGTTTTATGATGACCCC AAAGACCACCCATGTA
Hs_SLC34A1-std	ACGAAGCTCATCATCCAGCTGGACGAGTCTGTGATAAC CAGCATTGCCACTGGTGATGAGTCCCTGAGGAACCACA GTCTCATCCAGATCTGGTGCCACCCAGACTCCTTACAG CAAAA
Hs_SLC34A2-std	CCAAAACTGGACCATGAAGAATGTGACCTACAAGGAG AACATCGCCAAATGCCAGCATATCTTTGTGAATTTCCA CCTCCCGGATCTTGCTGTGGGC
Hs_SLC34A3-std	CTCATCGTGCAGTTGGACTCCGACATGATCATGAGCAG TGCCACAGGCAACGCCACTAACAGCAGTCTCATTAAGC ACTGGTGCGGCACCACGGGGCAGCCGACCCAGGAGAA CAGCAGC
Hs_XPR1-std	AATTTGTCTCATCAACATCTCTTTGAGATTGCTGGATTC CTCGGGATATTGTGGTGCCTGAGCCTTCTGGCATGCTTC TTTGCTCCAATTAGTGTCATCCCCACATATGT

Supplementary Table 9. Sequences of oligonucleotide primers used in qPCR with SYBR Green (Results are shown in Supplementary Fig. 6).

Primer Name	Sequences (5′→3′)
XPR1-F	ATCTCGTGGAGCAGATTGGC
XPR1-R	GGGGACACGTAAACGCTTCA
GAPDH-F	CACTAGGCGCTCACTGTTCT
GAPDH-R	GACCAAATCCGTTGACTCCG

Supplementary Methods

Characterisation of SC-919

SC-919, a single isomer, was prepared via stereoselectivity according to our developed method [1]. The absolute configuration of SC-919 was determined using X-ray crystallography; CCDC (Deposition Number) 2075338.

Kinase selectivity assay (kinome-wide selectivity profile of SC-919)

The kinome-wide selectivity profile of SC-919 was analysed using the KINOMEscan technology provided by DiscoveRx [2]. For most of the assays, kinase-tagged T7 phage strains were grown in parallel in 24-well blocks in an *E. coli* host derived from the BL21 strain. *E. coli* cells were grown until the log phase and infected with T7 phage from a frozen stock (multiplicity of infection = 0.4) and incubated with shaking at 32 °C until lysis occurred (90–150 min). The lysates were centrifuged (6,000 × *g*) and filtered (0.2 µm) to remove the cell debris. The remaining kinases were expressed in HEK293 cells and subsequently tagged with DNA for qPCR detection. Streptavidin-coated magnetic beads were treated with biotinylated small molecule ligands for 30 min at 22–26 °C to generate affinity resins for kinase assays. The liganded beads were blocked with excess biotin and washed with blocking buffer (SeaBlock [Pierce], 1 % BSA, 0.05 % Tween 20, 1 mM DTT) to remove the unbound ligand and to reduce the non-specific binding of phages. The binding reactions were assembled by mixing the kinases, liganded affinity beads, and test compounds in 1× binding buffer (20 % SeaBlock, 0.17× PBS, 0.05 % Tween 20, 6 mM DTT). Test compounds were prepared as 40× stocks in 100 % DMSO and were directly diluted into the assay mixture. All the reactions were performed in 384-well polypropylene plates at a final volume of 0.02 mL. The assay plates were incubated at 22–26 °C with shaking for 1 h, and the affinity beads were washed with wash buffer (1× PBS, 0.05 % Tween 20). The beads were resuspended in elution buffer (1x PBS, 0.05 % Tween 20, 0.5 µM non-biotinylated affinity ligand) and incubated at 22−26 °C with shaking for 30 min. The concentrations of kinases in the eluates were measured using qPCR. Detailed information is available at the Eurofin DiscoveRx website (https://www.discoverx.com/).

Qualification for the measurement of $InsP_6$ and $InsP_7$

Preparation of SC-919-treated sample for quality control (QC) sample

Cells (293, HAP1 wild-type) were treated with the 1 µM of SC-919 for 4 h, and were then immediately treated with trypsin and 10 % FCS-supplemented medium containing similar concentrations of SC-919. The cells were collected and centrifuged (2,000 × *g*, 1 min) and the supernatant was removed. The animals were used for *in vivo* experiments when they were 10 weeks old (Sprague-Dawley (SD) rats). SC-919 (10 mg/kg) was administered, and after 6 h, the rats were anesthetised with isoflurane (1–5 % v/v) and sacrificed by exsanguination to collect tissue samples. The cell and tissue samples were placed at −80 °C until required for analysis. The frozen tissue or cell samples were mixed with 3.6 % perchloric acid (v/v, prepared in distilled water [Nippon Gene]) (prepared using 0.2 and 0.3 mL of 3.6 % PCA per cell sample [1×10^6 and 4×10^6 cells for 293 and HAP1 wild-type cells, respectively] and 100 mg tissue samples). The samples incubated on ice were subsequently immediately homogenised for 2.5–10 min with zirconia beads (5 mm) using a mechanical homogeniser (1,100 rpm; Shakemaster, Bio Medical Science, Japan) followed by a spin-down. Subsequently, one-third (v/v, against PCA) of 30 % (w/v) potassium chloride solution was mixed with the homogenised samples with vortexing.

Next, samples were centrifuged (20,000 × *g*, 5 min, 4°C) and were used for subsequent analyses in $InsP_6$ and $InsP_7$ measurements.

Method qualification for the determination of $InsP_6$ and $InsP_7$ levels in tissues and cells using LC/MS/MS

LC/MS/MS conditions for determination of $InsP_6$ [099107, Matrix Scientific] and $InsP_7$ are shown in the main text. Stock solutions were prepared by dissolving accurately weighted $InsP_6$ or $InsP_7$ in water to yield a concentration of 10 mg/mL for $InsP_6$, 1 mg/mL for $InsP_7$. These stock solutions were further diluted in water to prepare working solutions for calibration curve, quality control (QC) samples and dilution integrity QC (DIQC) samples. The concentrations of working solutions ranged from 600 ng/mL to 600,000 ng/mL for $InsP_6$, 3 ng/mL to 30,000 ng/mL for $InsP_7$. Rat blank plasma was mixed with 3.6 % perchloric acid (v/v, prepared in distilled water) (prepared using 0.3 mL of 3.6 % PCA per 0.1 mL rat blank plasma). Subsequently, one-third (v/v, against PCA) of 30 % (w/v) potassium chloride solution was mixed with the mixed rat blank plasma with vortexing. Next, the mixtures were centrifuged (20,000 × *g*, 5 min, 4°C). The supernatant (PCA/potassium chloride-treated plasma) was used as blank matrix for preparation of calibration standard, and rat blank plasma was used as a surrogate matrix for tissues and Cells. Calibration standards for $InsP_6$ or $InsP_7$ were prepared by adding 10 µL of working solution to 150 µL of PCA/potassium chloride-treated plasma. QC samples in SC-919-treated sample for $InsP_6$ or $InsP_7$ were prepared by adding 10 µL of working solution to 150 µL of prepared SC-919-treated cell or tissue samples (see the section of "Preparation of SC-919-treated sample for quality control (QC) sample") at low (LQC), medium (MQC) and high (HQC) levels. Dilution integrity QC samples (DIQC) for $InsP_7$ in kidney were prepared by adding 8 µL of working solution at 30,000 ng/mL to 592 µL of prepared SC-919-treated kidney samples (see section "Preparation of SC-919-treated sample for quality control (QC) sample") at 400 ng/mL. After mixing, the mixtures were diluted with PCA/potassium chloride-treated plasma to 40 ng/mL (diluted DIQC sample). These calibration standards, QC and diluted DIQC samples were spiked with internal standard solution (20 µL, 500 ng/mL $InsP_6$-d_6 as an internal standard, 2,500 ng/mL *N, N, N', N'* – ethylenediaminetetrakis (methylenephosphonic acid) [E0393, Tokyo Chemical Industry]). After mixing, the mixtures were centrifuged (20,000 × *g*, 5 min, 4°C), and the supernatant was subjected to ultrafiltration (15,000 × *g*, 30 min, 4°C; Amicon Ultra-0.5 Centrifugal Filter Unit, 3 kDa Molecular Weight Cutoff [UFC5003, Merck KGaA]). The filtrates (96 µL) were mixed with 40 µL of water/hexylamine [H0134, Tokyo Chemical Industry]/acetic acid [01021-00, Kanto Chemical] (volume ratio, 44:3:3, respectively), then subjected to LC-MS/MS. Injection volume for determination of $InsP_6$ and $InsP_7$ was 2 µL and 25 µL, respectively.

The accuracy and precision for $InsP_6$ and $InsP_7$ were evaluated by analysing triplicates of QC samples. The results are shown in Supplementary Table 2 (calibration curve), Supplementary Table 3 and Supplementary Table 4 (accuracy and precision), and Supplementary Fig. 3 and Supplementary Fig. 4 (representative chromatogram of LQC).

Method for Supplementary Fig. 6

Preparation of protein samples for western blot analysis

Wild-type and *XPR1* KO HAP1 cells were cultured (200,000 cells/well) in 12-well culture plates with IMDM containing 10 % FCS. As an XPR1-positive control, the human *XPR1*-expressing vector was introduced into *XPR1* KO cells using the Xfect reagent following the procedure

described in the Methods section. The culture medium was replaced the next day, and the cells were cultured for one more day. The culture medium was then removed, and the cells were treated with M-PER™ Mammalian Protein Extraction Reagent (500 µL) containing the protease inhibitor cocktail (cOmplete™ ULTRA) by shaking the plate for 10 min at 22–26 °C. Samples were collected and centrifuged (12,000 × *g*, 1 min, 4 °C). The supernatant (250 µL) was mixed with 4× Laemmli Sample Buffer (250 µL, Bio-Rad) and 10 M urea solution (500 µL, final 5 M) in 2-mL tubes. The samples were properly mixed for 8 h at 22–26 °C using a rotator and used for western blot analysis. We used 10 (HAP1 wild-type and *XPR1* KO cells) and 2 µL (*XPR1*-overexpressed *XPR1* KO cells) samples for loading.

Antibodies

The antibodies used for western blot analysis were as follows: XPR1 (1:1,000, HPA016557, Atlas Antibodies), HRP-conjugated beta-actin (1:2,000, PM053-7, MEDICAL & BIOLOGICAL LABORATORIES CO., LTD.), and Peroxidase-AffiniPure Goat anti-Rabbit IgG (H+L) (1:10,000, 111-035-144, Jackson ImmunoResearch).

SDS-PAGE and immunoblotting

The proteins were separated using SDS/PAGE (TGX gel, Bio-Rad) and transferred onto a PVDF membrane (Bio-Rad). The membrane was blocked in 5 % BSA and incubated for 18 h with the primary antibodies and subsequently with the respective secondary antibodies. Thereafter, the blot was developed using the ECL reagent (GE Healthcare) and immunoreactive signals were detected using the LAS-3000 imaging system (FUJIFILM Wako).

Preparation of total RNA and cDNA

Tissue samples were dissected and immediately frozen on dry ice. The tissue or cell samples were homogenised in QIAzol Lysis Reagent (QIAGEN), and total RNA was isolated using RNeasy kit purification (QIAGEN). First-strand cDNA synthesised was performed using the ReverTra Ace® qPCR RT Master Mix (Toyobo).

Measurement of mRNA levels

To determine the relative mRNA expression levels, a quantitative polymerase chain reaction was performed using the TaqMan gene expression assay (*Nphs2*, Rn00709834_m1; *Cyp27b1*, Rn01647147_g1; Rn28s as an internal control, Rn03034784_g1). The expression level relative to that in the vehicle-treated group was calculated using the comparative CT ($2^{-\Delta\Delta CT}$) method (User Bulletin #2, Applied Biosystems). For the comparative CT method, the ΔCT value was determined by subtracting the average of the CT value for the internal control from the average of the CT value for the target. The absolute copy number of the target gene was determined using a standard curve generated by amplifying known concentrations of synthetic oligonucleotides. The oligonucleotide primers, dual-labelled (FAM-TAMRA) oligonucleotide probes, and standard oligonucleotides were synthesised by Sigma-Aldrich (Supplementary Table 8 and Supplementary Table 9). Amplification was performed on an ABI PRISM 7900HT Sequence Detector (Thermo Fisher Scientific) using the EXPRESS qPCR Supermix (Thermo Fisher Scientific) or THUNDERBIRD® qPCR Mix (Toyobo) as recommended by the manufacturers.

Treatment of cells with test compounds and determination of cellular protein levels

Cultured cells (293, HAP1 wild-type, HAP1 *XPR1* KO) were treated with the indicated concentration of the compound for 4 h. The protein was extracted from the cells using the M-PER protein extraction reagent, and protein levels were determined using the Pierce® 660 nm protein assay (Thermo Fisher Scientific).

Supplementary References

1. Terao, Y.*, et al.* WO2018182051 IP6K INHIBITORS. (2018).
2. Fabian, M.A.*, et al.* A small molecule-kinase interaction map for clinical kinase inhibitors. *Nat Biotechnol* **23**, 329-336 (2005).
