# Supplementary material for: The enzymatic activity of inositol hexakisphosphate kinase controls circulating phosphate in mammals
Source: Nat Commun. 2021 Aug 11;12:4847. doi: 10.1038/s41467-021-24934-8 (PMC8358040; doi:10.1038/s41467-021-24934-8)
Supplement: Supplementary file 3 — Reporting Summary [file 41467_2021_24934_MOESM3_ESM.pdf]

nature research

Corresponding author(s): Yusuke Moritoh

Last updated by author(s): Jun 23, 2021

Reporting Summary

Nature Research wishes to improve the reproducibility of the work that we publish. This form provides structure for consistency and transparency in reporting. For further information on Nature Research policies, see our Editorial Policies and the Editorial Policy Checklist.

Statistics

For all statistical analyses, confirm that the following items are present in the figure legend, table legend, main text, or Methods section.

n/a	Confirmed	
☐	☒	The exact sample size (*n*) for each experimental group/condition, given as a discrete number and unit of measurement
☐	☒	A statement on whether measurements were taken from distinct samples or whether the same sample was measured repeatedly
☐	☒	The statistical test(s) used AND whether they are one- or two-sided<br>*Only common tests should be described solely by name; describe more complex techniques in the Methods section.*
☒	☐	A description of all covariates tested
☐	☒	A description of any assumptions or corrections, such as tests of normality and adjustment for multiple comparisons
☐	☒	A full description of the statistical parameters including central tendency (e.g. means) or other basic estimates (e.g. regression coefficient) AND variation (e.g. standard deviation) or associated estimates of uncertainty (e.g. confidence intervals)
☐	☒	For null hypothesis testing, the test statistic (e.g. *F*, *t*, *r*) with confidence intervals, effect sizes, degrees of freedom and *P* value noted<br>*Give P values as exact values whenever suitable.*
☒	☐	For Bayesian analysis, information on the choice of priors and Markov chain Monte Carlo settings
☒	☐	For hierarchical and complex designs, identification of the appropriate level for tests and full reporting of outcomes
☒	☐	Estimates of effect sizes (e.g. Cohen's *d*, Pearson's *r*), indicating how they were calculated

Our web collection on statistics for biologists contains articles on many of the points above.

Software and code

Policy information about availability of computer code

Data collection	Data were collected using Analyst 1.6.3 (LC/MS/MS measurement), ImageReader LAS-3000 2.21 (Western blotting andg gel imaging), Gen5 2.01.14 colorimetric assay), and EnVison Manager 1.13.3009.1401 (chemiluminescence, fluorometric, and colorimetric assays).
Data analysis	The data were analysed using Microsoft Excel for Office 365, GraphPad Prism 7, and WinROOF2015.

For manuscripts utilizing custom algorithms or software that are central to the research but not yet described in published literature, software must be made available to editors and reviewers. We strongly encourage code deposition in a community repository (e.g. GitHub). See the Nature Research guidelines for submitting code & software for further information.

Data

Policy information about availability of data

All manuscripts must include a data availability statement. This statement should provide the following information, where applicable:

- Accession codes, unique identifiers, or web links for publicly available datasets
- A list of figures that have associated raw data
- A description of any restrictions on data availability

All data that support the findings of this study are included in the manuscript or will be available from the authors upon reasonable request. Source data are provided in this paper.

Field-specific reporting

Please select the one below that is the best fit for your research. If you are not sure, read the appropriate sections before making your selection.

☒ Life sciences ☐ Behavioural & social sciences ☐ Ecological, evolutionary & environmental sciences

For a reference copy of the document with all sections, see nature.com/documents/nr-reporting-summary-flat.pdf

Life sciences study design

All studies must disclose on these points even when the disclosure is negative.

Sample size	Sample size was determined based on the preliminary experiments conducted in advance.
Data exclusions	Data were not excluded except in cases of technical error.
Replication	Reliability of the data was ensured by using more than three biological replicates. All attempts at replication of experimental findings more than one time were reliably reproduced.
Randomization	In vitro and in vivo samples were randomly allocated to groups.
Blinding	Blinding was not relevant as the same investigators conducted the experiments, collected samples, and analysed data.

Reporting for specific materials, systems and methods

We require information from authors about some types of materials, experimental systems and methods used in many studies. Here, indicate whether each material, system or method listed is relevant to your study. If you are not sure if a list item applies to your research, read the appropriate section before selecting a response.

Materials & experimental systems

n/a	Involved in the study	
☐	☒	Antibodies
☐	☒	Eukaryotic cell lines
☒	☐	Palaeontology and archaeology
☐	☒	Animals and other organisms
☒	☐	Human research participants
☒	☐	Clinical data
☒	☐	Dual use research of concern

Methods

n/a	Involved in the study	
☒	☐	ChIP-seq
☒	☐	Flow cytometry
☒	☐	MRI-based neuroimaging

Antibodies

Antibodies used	The antibodies used for western blot analysis were as follows: XPR1 (1/1000, HPA016557, Atlas Antibodies), HRP-conjugated beta-actin (1/2000, PM053-7, MEDICAL & BIOLOGICAL LABORATORIES CO., LTD.), and Peroxidase-AffiniPure Goat anti-Rabbit IgG (H+L) (1/10000, 111-035-144, Jackson ImmunoResearch).
Validation	HPA016557 was validated using XPR1 KO cells. PM053-7 was validated by MEDICAL & BIOLOGICAL LABORATORIES CO., LTD.

Eukaryotic cell lines

Policy information about cell lines

Cell line source(s)	HEK293 cells were obtained from the European Collection of Authenticated Cell Cultures (85120602). Parental (wild-type, C631) and XPR1 KO HAP1 cells (HZGHC004238c008) were obtained from Horizon Discovery. Sf9 cells were obtained from Thermo Fisher Scientific.
Authentication	The cell lines have been authenticated by the suppliers.
Mycoplasma contamination	All cell lines used were tested negative for mycoplasma contamination.
Commonly misidentified lines (See ICLAC register)	No commonly misidentified cell lines were used.

Animals and other organisms

Policy information about studies involving animals; ARRIVE guidelines recommended for reporting animal research

Laboratory animals	Male SD rats were obtained from CLEA Japan, Inc. The experiment using male cynomolgus monkeys was performed in Hamamatsu Pharma Research, Inc.(Hamamatsu, Shizuoka, Japan), which is accredited by the American Association for Accreditation of Laboratory Animal Care. Ages were included in main text.
Wild animals	Studies did not include wild animals.
Field-collected samples	Studies did not include samples collected from the field.
Ethics oversight	The protocols for the care of animals and experiments were approved by the Institutional Animal Care and Use Committee at Shonan Health Innovation Park, which is accredited by the American Association for Accreditation of Laboratory Animal Care.

Note that full information on the approval of the study protocol must also be provided in the manuscript.
